# Supplementary material for: Modeling glioblastoma heterogeneity as a dynamic network of cell states
Source: Mol Syst Biol. 2021 Sep 16;17(9):e10105. doi: 10.15252/msb.202010105 (PMC8444284; doi:10.15252/msb.202010105)
Supplement: Supplementary file 6 — Source Data for Figure 5 [file MSB-17-e10105-s004.zip › Figure5A_sourcedata/GSEA_3071/nosigdb_stateII.GseaPreranked.1622795151171/RICHARDS_NOWAKOWSKI_SCIENCE_2017_MGE_IPC1_UPREG.html]

Details for gene set RICHARDS\_NOWAKOWSKI\_SCIENCE\_2017\_MGE\_IPC1\_UPREG[GSEA]

|  || Dataset | state23071 |
| Phenotype | NoPhenotypeAvailable |
| Upregulated in class | na\_pos |
| GeneSet | RICHARDS\_NOWAKOWSKI\_SCIENCE\_2017\_MGE\_IPC1\_UPREG |
| Enrichment Score (ES) | 0.4818495 |
| Normalized Enrichment Score (NES) | 3.6278815 |
| Nominal p-value | 0.0 |
| FDR q-value | 0.0 |
| FWER p-Value | 0.0 |
Table: GSEA Results Summary

  

Fig 1: Enrichment plot: RICHARDS\_NOWAKOWSKI\_SCIENCE\_2017\_MGE\_IPC1\_UPREG      
 Profile of the Running ES Score & Positions of GeneSet Members on the Rank Ordered List

  

| PROBE | GENE SYMBOL | GENE\_TITLE | RANK IN GENE LIST | RANK METRIC SCORE | RUNNING ES | CORE ENRICHMENT || 1 | HIST1H1D |  |  | 0 | 2.323 | 0.0089 | Yes |
| 2 | HIST1H4C |  |  | 2 | 1.902 | 0.0160 | Yes |
| 3 | RRM2 |  |  | 3 | 1.857 | 0.0231 | Yes |
| 4 | ANLN |  |  | 4 | 1.760 | 0.0299 | Yes |
| 5 | HIST1H1B |  |  | 7 | 1.669 | 0.0358 | Yes |
| 6 | TYMS |  |  | 8 | 1.665 | 0.0422 | Yes |
| 7 | CDK1 |  |  | 14 | 1.540 | 0.0467 | Yes |
| 8 | DIAPH3 |  |  | 15 | 1.531 | 0.0526 | Yes |
| 9 | ZWINT |  |  | 16 | 1.510 | 0.0584 | Yes |
| 10 | HJURP |  |  | 17 | 1.490 | 0.0642 | Yes |
| 11 | BRCA2 |  |  | 22 | 1.445 | 0.0686 | Yes |
| 12 | UBE2C |  |  | 23 | 1.438 | 0.0742 | Yes |
| 13 | CENPU |  |  | 24 | 1.416 | 0.0796 | Yes |
| 14 | RAD51AP1 |  |  | 25 | 1.384 | 0.0849 | Yes |
| 15 | CENPK |  |  | 26 | 1.382 | 0.0903 | Yes |
| 16 | FBXO5 |  |  | 30 | 1.359 | 0.0947 | Yes |
| 17 | USP1 |  |  | 31 | 1.357 | 0.0999 | Yes |
| 18 | ORC6 |  |  | 39 | 1.308 | 0.1030 | Yes |
| 19 | CDCA5 |  |  | 41 | 1.289 | 0.1077 | Yes |
| 20 | AURKB |  |  | 44 | 1.276 | 0.1121 | Yes |
| 21 | CCNA2 |  |  | 53 | 1.230 | 0.1147 | Yes |
| 22 | NEIL3 |  |  | 54 | 1.228 | 0.1194 | Yes |
| 23 | TICRR |  |  | 55 | 1.227 | 0.1241 | Yes |
| 24 | SKA3 |  |  | 57 | 1.215 | 0.1285 | Yes |
| 25 | HIST1H1C |  |  | 60 | 1.206 | 0.1326 | Yes |
| 26 | MAD2L1 |  |  | 61 | 1.199 | 0.1372 | Yes |
| 27 | UBE2T |  |  | 62 | 1.192 | 0.1418 | Yes |
| 28 | ATAD2 |  |  | 63 | 1.189 | 0.1464 | Yes |
| 29 | SPC25 |  |  | 68 | 1.156 | 0.1497 | Yes |
| 30 | MELK |  |  | 69 | 1.145 | 0.1541 | Yes |
| 31 | TOP2A |  |  | 72 | 1.132 | 0.1580 | Yes |
| 32 | KIF2C |  |  | 73 | 1.132 | 0.1623 | Yes |
| 33 | UHRF1 |  |  | 79 | 1.113 | 0.1652 | Yes |
| 34 | FAM111A |  |  | 80 | 1.112 | 0.1695 | Yes |
| 35 | GMNN |  |  | 83 | 1.108 | 0.1732 | Yes |
| 36 | CEP55 |  |  | 87 | 1.096 | 0.1767 | Yes |
| 37 | KIFC1 |  |  | 88 | 1.094 | 0.1809 | Yes |
| 38 | HIST1H1E |  |  | 90 | 1.088 | 0.1848 | Yes |
| 39 | SKA1 |  |  | 91 | 1.078 | 0.1889 | Yes |
| 40 | VRK1 |  |  | 93 | 1.074 | 0.1928 | Yes |
| 41 | NDC80 |  |  | 94 | 1.074 | 0.1969 | Yes |
| 42 | LRR1 |  |  | 98 | 1.060 | 0.2002 | Yes |
| 43 | TMPO |  |  | 99 | 1.056 | 0.2042 | Yes |
| 44 | NCAPG |  |  | 100 | 1.056 | 0.2083 | Yes |
| 45 | MYBL1 |  |  | 102 | 1.043 | 0.2120 | Yes |
| 46 | MND1 |  |  | 104 | 1.036 | 0.2158 | Yes |
| 47 | MKI67 |  |  | 107 | 1.031 | 0.2192 | Yes |
| 48 | STIL |  |  | 108 | 1.031 | 0.2231 | Yes |
| 49 | TPX2 |  |  | 111 | 1.025 | 0.2265 | Yes |
| 50 | CDCA8 |  |  | 113 | 1.018 | 0.2302 | Yes |
| 51 | KIF11 |  |  | 115 | 1.014 | 0.2338 | Yes |
| 52 | NCAPH |  |  | 116 | 1.013 | 0.2377 | Yes |
| 53 | SMC2 |  |  | 123 | 1.005 | 0.2400 | Yes |
| 54 | NUF2 |  |  | 125 | 1.004 | 0.2436 | Yes |
| 55 | DEPDC1 |  |  | 126 | 0.999 | 0.2474 | Yes |
| 56 | CKS1B |  |  | 128 | 0.999 | 0.2510 | Yes |
| 57 | TACC3 |  |  | 130 | 0.992 | 0.2545 | Yes |
| 58 | CENPL |  |  | 132 | 0.989 | 0.2581 | Yes |
| 59 | KIF20B |  |  | 133 | 0.989 | 0.2619 | Yes |
| 60 | MGME1 |  |  | 135 | 0.988 | 0.2654 | Yes |
| 61 | FBN1 |  |  | 143 | 0.972 | 0.2672 | Yes |
| 62 | ESCO2 |  |  | 144 | 0.971 | 0.2710 | Yes |
| 63 | CKAP2L |  |  | 148 | 0.959 | 0.2738 | Yes |
| 64 | NCAPG2 |  |  | 159 | 0.937 | 0.2748 | Yes |
| 65 | KIF23 |  |  | 161 | 0.936 | 0.2781 | Yes |
| 66 | SHCBP1 |  |  | 164 | 0.933 | 0.2811 | Yes |
| 67 | RTKN2 |  |  | 166 | 0.931 | 0.2844 | Yes |
| 68 | GTSE1 |  |  | 167 | 0.929 | 0.2880 | Yes |
| 69 | CDCA2 |  |  | 171 | 0.922 | 0.2908 | Yes |
| 70 | PIM1 |  |  | 184 | 0.896 | 0.2910 | Yes |
| 71 | ZNF850 |  |  | 187 | 0.888 | 0.2938 | Yes |
| 72 | ADAMTS6 |  |  | 191 | 0.884 | 0.2964 | Yes |
| 73 | FAM83D |  |  | 197 | 0.876 | 0.2984 | Yes |
| 74 | SPDL1 |  |  | 199 | 0.874 | 0.3015 | Yes |
| 75 | C21orf58 |  |  | 201 | 0.873 | 0.3046 | Yes |
| 76 | PLK4 |  |  | 205 | 0.870 | 0.3072 | Yes |
| 77 | H2AFZ |  |  | 213 | 0.864 | 0.3086 | Yes |
| 78 | PBK |  |  | 214 | 0.864 | 0.3119 | Yes |
| 79 | CEP152 |  |  | 217 | 0.860 | 0.3147 | Yes |
| 80 | CDCA3 |  |  | 219 | 0.858 | 0.3177 | Yes |
| 81 | NET1 |  |  | 220 | 0.857 | 0.3210 | Yes |
| 82 | BUB1B |  |  | 223 | 0.851 | 0.3238 | Yes |
| 83 | ASPM |  |  | 231 | 0.842 | 0.3251 | Yes |
| 84 | EZH2 |  |  | 233 | 0.839 | 0.3281 | Yes |
| 85 | MIS18A |  |  | 239 | 0.833 | 0.3299 | Yes |
| 86 | DBF4 |  |  | 240 | 0.832 | 0.3331 | Yes |
| 87 | RACGAP1 |  |  | 241 | 0.831 | 0.3363 | Yes |
| 88 | PRC1 |  |  | 242 | 0.830 | 0.3395 | Yes |
| 89 | TUBB6 |  |  | 244 | 0.828 | 0.3424 | Yes |
| 90 | LMNB1 |  |  | 251 | 0.824 | 0.3440 | Yes |
| 91 | KIF18B |  |  | 254 | 0.815 | 0.3466 | Yes |
| 92 | ACTN1 |  |  | 256 | 0.813 | 0.3494 | Yes |
| 93 | DNA2 |  |  | 263 | 0.797 | 0.3509 | Yes |
| 94 | DKC1 |  |  | 268 | 0.789 | 0.3528 | Yes |
| 95 | CENPI |  |  | 269 | 0.788 | 0.3559 | Yes |
| 96 | TUBB4B |  |  | 271 | 0.784 | 0.3586 | Yes |
| 97 | HMGB2 |  |  | 276 | 0.780 | 0.3605 | Yes |
| 98 | TUBA1C |  |  | 277 | 0.779 | 0.3635 | Yes |
| 99 | SMC4 |  |  | 279 | 0.777 | 0.3662 | Yes |
| 100 | CENPE |  |  | 284 | 0.775 | 0.3681 | Yes |
| 101 | TEX30 |  |  | 287 | 0.774 | 0.3706 | Yes |
| 102 | RFWD3 |  |  | 290 | 0.769 | 0.3730 | Yes |
| 103 | BARD1 |  |  | 295 | 0.760 | 0.3748 | Yes |
| 104 | DEK |  |  | 302 | 0.754 | 0.3761 | Yes |
| 105 | CENPA |  |  | 303 | 0.751 | 0.3790 | Yes |
| 106 | CENPO |  |  | 307 | 0.747 | 0.3811 | Yes |
| 107 | KIF4A |  |  | 309 | 0.746 | 0.3837 | Yes |
| 108 | CKS2 |  |  | 312 | 0.745 | 0.3860 | Yes |
| 109 | PRR11 |  |  | 314 | 0.743 | 0.3886 | Yes |
| 110 | CENPN |  |  | 320 | 0.737 | 0.3901 | Yes |
| 111 | CCNF |  |  | 321 | 0.737 | 0.3929 | Yes |
| 112 | ANP32B |  |  | 325 | 0.734 | 0.3949 | Yes |
| 113 | CENPW |  |  | 330 | 0.727 | 0.3966 | Yes |
| 114 | SPC24 |  |  | 340 | 0.719 | 0.3970 | Yes |
| 115 | ARHGAP11A |  |  | 346 | 0.712 | 0.3984 | Yes |
| 116 | LMNB2 |  |  | 352 | 0.709 | 0.3997 | Yes |
| 117 | AURKA |  |  | 360 | 0.705 | 0.4006 | Yes |
| 118 | DTYMK |  |  | 364 | 0.701 | 0.4025 | Yes |
| 119 | TTK |  |  | 365 | 0.701 | 0.4052 | Yes |
| 120 | CENPJ |  |  | 376 | 0.695 | 0.4051 | Yes |
| 121 | KIF14 |  |  | 378 | 0.693 | 0.4075 | Yes |
| 122 | PARPBP |  |  | 382 | 0.688 | 0.4094 | Yes |
| 123 | MTFR2 |  |  | 386 | 0.686 | 0.4112 | Yes |
| 124 | KIF18A |  |  | 392 | 0.683 | 0.4125 | Yes |
| 125 | EXOSC9 |  |  | 399 | 0.679 | 0.4135 | Yes |
| 126 | KIF15 |  |  | 404 | 0.676 | 0.4150 | Yes |
| 127 | CCDC34 |  |  | 411 | 0.672 | 0.4160 | Yes |
| 128 | TTF2 |  |  | 413 | 0.671 | 0.4183 | Yes |
| 129 | SAE1 |  |  | 418 | 0.669 | 0.4198 | Yes |
| 130 | KPNA2 |  |  | 420 | 0.669 | 0.4221 | Yes |
| 131 | FOXM1 |  |  | 423 | 0.667 | 0.4241 | Yes |
| 132 | RAN |  |  | 424 | 0.667 | 0.4267 | Yes |
| 133 | SMC3 |  |  | 425 | 0.667 | 0.4292 | Yes |
| 134 | PHGDH |  |  | 429 | 0.664 | 0.4310 | Yes |
| 135 | INCENP |  |  | 432 | 0.660 | 0.4330 | Yes |
| 136 | BUB3 |  |  | 441 | 0.657 | 0.4333 | Yes |
| 137 | PKP4 |  |  | 444 | 0.654 | 0.4353 | Yes |
| 138 | PIF1 |  |  | 445 | 0.653 | 0.4378 | Yes |
| 139 | NCAPD2 |  |  | 457 | 0.647 | 0.4373 | Yes |
| 140 | HMMR |  |  | 475 | 0.639 | 0.4352 | Yes |
| 141 | FADS1 |  |  | 498 | 0.629 | 0.4317 | Yes |
| 142 | LMO7 |  |  | 499 | 0.629 | 0.4341 | Yes |
| 143 | RHEB |  |  | 500 | 0.629 | 0.4365 | Yes |
| 144 | BIRC5 |  |  | 505 | 0.628 | 0.4379 | Yes |
| 145 | NOM1 |  |  | 508 | 0.627 | 0.4398 | Yes |
| 146 | ECT2 |  |  | 510 | 0.626 | 0.4419 | Yes |
| 147 | IQGAP3 |  |  | 516 | 0.621 | 0.4429 | Yes |
| 148 | GEN1 |  |  | 517 | 0.620 | 0.4453 | Yes |
| 149 | SNAPC1 |  |  | 520 | 0.617 | 0.4471 | Yes |
| 150 | NDC1 |  |  | 521 | 0.617 | 0.4495 | Yes |
| 151 | HAUS8 |  |  | 527 | 0.613 | 0.4505 | Yes |
| 152 | C5orf34 |  |  | 533 | 0.611 | 0.4515 | Yes |
| 153 | DDX10 |  |  | 537 | 0.609 | 0.4531 | Yes |
| 154 | PPM1G |  |  | 539 | 0.609 | 0.4551 | Yes |
| 155 | BTG3 |  |  | 540 | 0.607 | 0.4575 | Yes |
| 156 | DDX39A |  |  | 542 | 0.605 | 0.4595 | Yes |
| 157 | NPM1 |  |  | 544 | 0.605 | 0.4616 | Yes |
| 158 | RCC1 |  |  | 549 | 0.603 | 0.4628 | Yes |
| 159 | C18orf54 |  |  | 550 | 0.603 | 0.4651 | Yes |
| 160 | MIS18BP1 |  |  | 574 | 0.588 | 0.4612 | Yes |
| 161 | CENPF |  |  | 575 | 0.588 | 0.4635 | Yes |
| 162 | CCNB1 |  |  | 579 | 0.587 | 0.4649 | Yes |
| 163 | CEP135 |  |  | 598 | 0.578 | 0.4623 | Yes |
| 164 | EXOSC8 |  |  | 604 | 0.576 | 0.4632 | Yes |
| 165 | TUBG1 |  |  | 617 | 0.570 | 0.4621 | Yes |
| 166 | CKAP2 |  |  | 619 | 0.569 | 0.4640 | Yes |
| 167 | CDC25C |  |  | 626 | 0.568 | 0.4646 | Yes |
| 168 | ADCY3 |  |  | 630 | 0.566 | 0.4660 | Yes |
| 169 | SPAG5 |  |  | 642 | 0.555 | 0.4651 | Yes |
| 170 | NUSAP1 |  |  | 654 | 0.550 | 0.4643 | Yes |
| 171 | LSM6 |  |  | 657 | 0.550 | 0.4659 | Yes |
| 172 | FANCM |  |  | 659 | 0.549 | 0.4677 | Yes |
| 173 | CCT5 |  |  | 662 | 0.548 | 0.4693 | Yes |
| 174 | H2AFX |  |  | 671 | 0.544 | 0.4692 | Yes |
| 175 | MNS1 |  |  | 672 | 0.543 | 0.4713 | Yes |
| 176 | NUP107 |  |  | 675 | 0.539 | 0.4728 | Yes |
| 177 | TUBB |  |  | 678 | 0.538 | 0.4744 | Yes |
| 178 | CEP85 |  |  | 683 | 0.537 | 0.4753 | Yes |
| 179 | WEE1 |  |  | 685 | 0.536 | 0.4771 | Yes |
| 180 | CIT |  |  | 690 | 0.535 | 0.4781 | Yes |
| 181 | NUP205 |  |  | 705 | 0.531 | 0.4764 | Yes |
| 182 | DLGAP5 |  |  | 712 | 0.529 | 0.4768 | Yes |
| 183 | GPSM2 |  |  | 719 | 0.526 | 0.4772 | Yes |
| 184 | CCT6A |  |  | 725 | 0.525 | 0.4779 | Yes |
| 185 | C4orf46 |  |  | 733 | 0.521 | 0.4780 | Yes |
| 186 | CCDC18 |  |  | 735 | 0.520 | 0.4797 | Yes |
| 187 | RANGAP1 |  |  | 736 | 0.520 | 0.4817 | Yes |
| 188 | PHF19 |  |  | 745 | 0.518 | 0.4816 | Yes |
| 189 | ACTG1 |  |  | 755 | 0.514 | 0.4811 | Yes |
| 190 | BORA |  |  | 766 | 0.511 | 0.4804 | Yes |
| 191 | POLQ |  |  | 771 | 0.508 | 0.4813 | Yes |
| 192 | NUP62 |  |  | 777 | 0.505 | 0.4818 | Yes |
| 193 | CCDC150 |  |  | 804 | 0.496 | 0.4767 | No |
| 194 | AHI1 |  |  | 814 | 0.494 | 0.4762 | No |
| 195 | LIN54 |  |  | 823 | 0.490 | 0.4759 | No |
| 196 | SRSF10 |  |  | 831 | 0.489 | 0.4759 | No |
| 197 | CDC20 |  |  | 844 | 0.486 | 0.4746 | No |
| 198 | TUBA1B |  |  | 862 | 0.481 | 0.4718 | No |
| 199 | IKBIP |  |  | 876 | 0.477 | 0.4702 | No |
| 200 | NUP50 |  |  | 888 | 0.473 | 0.4690 | No |
| 201 | CEP57L1 |  |  | 901 | 0.471 | 0.4676 | No |
| 202 | DEPDC1B |  |  | 906 | 0.470 | 0.4683 | No |
| 203 | CDKN2D |  |  | 911 | 0.468 | 0.4691 | No |
| 204 | ITGB3BP |  |  | 935 | 0.462 | 0.4646 | No |
| 205 | LIN9 |  |  | 949 | 0.459 | 0.4629 | No |
| 206 | CDC7 |  |  | 971 | 0.451 | 0.4590 | No |
| 207 | KIF22 |  |  | 975 | 0.450 | 0.4599 | No |
| 208 | FANCD2 |  |  | 976 | 0.450 | 0.4616 | No |
| 209 | PRIMPOL |  |  | 978 | 0.449 | 0.4631 | No |
| 210 | PLK1 |  |  | 1009 | 0.442 | 0.4567 | No |
| 211 | NUP35 |  |  | 1020 | 0.438 | 0.4557 | No |
| 212 | TBC1D31 |  |  | 1050 | 0.428 | 0.4495 | No |
| 213 | DLEU2 |  |  | 1059 | 0.426 | 0.4490 | No |
| 214 | DARS2 |  |  | 1062 | 0.425 | 0.4501 | No |
| 215 | HYLS1 |  |  | 1065 | 0.425 | 0.4512 | No |
| 216 | ARHGAP11B |  |  | 1069 | 0.424 | 0.4520 | No |
| 217 | CHEK2 |  |  | 1073 | 0.424 | 0.4528 | No |
| 218 | PABPC1 |  |  | 1075 | 0.422 | 0.4542 | No |
| 219 | JADE1 |  |  | 1089 | 0.419 | 0.4523 | No |
| 220 | NEK2 |  |  | 1100 | 0.417 | 0.4512 | No |
| 221 | KCTD9 |  |  | 1104 | 0.416 | 0.4520 | No |
| 222 | CTDSPL2 |  |  | 1109 | 0.415 | 0.4525 | No |
| 223 | TDP1 |  |  | 1112 | 0.415 | 0.4536 | No |
| 224 | SRRT |  |  | 1121 | 0.414 | 0.4530 | No |
| 225 | CBX5 |  |  | 1132 | 0.412 | 0.4519 | No |
| 226 | UCP2 |  |  | 1138 | 0.410 | 0.4521 | No |
| 227 | BUB1 |  |  | 1145 | 0.408 | 0.4521 | No |
| 228 | TPRKB |  |  | 1168 | 0.403 | 0.4477 | No |
| 229 | POC5 |  |  | 1179 | 0.401 | 0.4465 | No |
| 230 | SCLT1 |  |  | 1201 | 0.397 | 0.4424 | No |
| 231 | SLC3A2 |  |  | 1203 | 0.396 | 0.4437 | No |
| 232 | MZT1 |  |  | 1219 | 0.393 | 0.4411 | No |
| 233 | PTMA |  |  | 1233 | 0.390 | 0.4391 | No |
| 234 | APOLD1 |  |  | 1239 | 0.389 | 0.4393 | No |
| 235 | FGFR1OP |  |  | 1254 | 0.386 | 0.4370 | No |
| 236 | ESPL1 |  |  | 1255 | 0.386 | 0.4385 | No |
| 237 | NUP98 |  |  | 1314 | 0.375 | 0.4243 | No |
| 238 | STK17B |  |  | 1324 | 0.374 | 0.4233 | No |
| 239 | ANP32E |  |  | 1334 | 0.371 | 0.4223 | No |
| 240 | DR1 |  |  | 1352 | 0.366 | 0.4191 | No |
| 241 | LDHA |  |  | 1374 | 0.361 | 0.4149 | No |
| 242 | ITGB1BP1 |  |  | 1382 | 0.360 | 0.4144 | No |
| 243 | EIF3D |  |  | 1405 | 0.357 | 0.4098 | No |
| 244 | SPATA5 |  |  | 1424 | 0.352 | 0.4063 | No |
| 245 | KNSTRN |  |  | 1458 | 0.346 | 0.3987 | No |
| 246 | EIF5A |  |  | 1461 | 0.346 | 0.3995 | No |
| 247 | RBM15 |  |  | 1467 | 0.345 | 0.3995 | No |
| 248 | AHCY |  |  | 1471 | 0.344 | 0.4000 | No |
| 249 | ECI2 |  |  | 1516 | 0.338 | 0.3895 | No |
| 250 | FUBP1 |  |  | 1519 | 0.337 | 0.3902 | No |
| 251 | CNTROB |  |  | 1528 | 0.335 | 0.3893 | No |
| 252 | TRERF1 |  |  | 1530 | 0.335 | 0.3904 | No |
| 253 | CLIC1 |  |  | 1552 | 0.331 | 0.3860 | No |
| 254 | CDC27 |  |  | 1562 | 0.330 | 0.3848 | No |
| 255 | TIMM10 |  |  | 1567 | 0.329 | 0.3850 | No |
| 256 | UTRN |  |  | 1615 | 0.321 | 0.3736 | No |
| 257 | CALM2 |  |  | 1625 | 0.320 | 0.3724 | No |
| 258 | DLEU1 |  |  | 1638 | 0.318 | 0.3704 | No |
| 259 | SF3B3 |  |  | 1651 | 0.316 | 0.3684 | No |
| 260 | ARHGAP19 |  |  | 1658 | 0.316 | 0.3679 | No |
| 261 | G3BP1 |  |  | 1661 | 0.315 | 0.3686 | No |
| 262 | HSPA2 |  |  | 1688 | 0.312 | 0.3628 | No |
| 263 | TMX1 |  |  | 1703 | 0.310 | 0.3602 | No |
| 264 | HMGB1 |  |  | 1720 | 0.308 | 0.3571 | No |
| 265 | ZMYM1 |  |  | 1737 | 0.306 | 0.3540 | No |
| 266 | RUVBL2 |  |  | 1779 | 0.301 | 0.3441 | No |
| 267 | SAV1 |  |  | 1792 | 0.299 | 0.3420 | No |
| 268 | CNTRL |  |  | 1830 | 0.294 | 0.3332 | No |
| 269 | ILF2 |  |  | 1833 | 0.294 | 0.3337 | No |
| 270 | SRSF3 |  |  | 1834 | 0.294 | 0.3349 | No |
| 271 | AFG3L2 |  |  | 1840 | 0.293 | 0.3347 | No |
| 272 | STK39 |  |  | 1871 | 0.289 | 0.3277 | No |
| 273 | UBAP2 |  |  | 1877 | 0.289 | 0.3274 | No |
| 274 | ODF2 |  |  | 1887 | 0.287 | 0.3261 | No |
| 275 | QSER1 |  |  | 1922 | 0.282 | 0.3180 | No |
| 276 | GDI2 |  |  | 1928 | 0.282 | 0.3178 | No |
| 277 | NUP37 |  |  | 1929 | 0.281 | 0.3189 | No |
| 278 | PSMB2 |  |  | 1950 | 0.279 | 0.3145 | No |
| 279 | RPL22L1 |  |  | 1966 | 0.277 | 0.3116 | No |
| 280 | OXA1L |  |  | 1969 | 0.277 | 0.3121 | No |
| 281 | RBM8A |  |  | 1971 | 0.276 | 0.3129 | No |
| 282 | FAM122B |  |  | 2009 | 0.271 | 0.3040 | No |
| 283 | SKA2 |  |  | 2061 | 0.266 | 0.2912 | No |
| 284 | CPSF3 |  |  | 2081 | 0.264 | 0.2871 | No |
| 285 | MPHOSPH9 |  |  | 2082 | 0.264 | 0.2881 | No |
| 286 | CEP192 |  |  | 2109 | 0.262 | 0.2821 | No |
| 287 | LDLR |  |  | 2128 | 0.260 | 0.2783 | No |
| 288 | CKAP5 |  |  | 2135 | 0.258 | 0.2777 | No |
| 289 | MXD3 |  |  | 2156 | 0.256 | 0.2733 | No |
| 290 | PIGK |  |  | 2159 | 0.256 | 0.2737 | No |
| 291 | ZCCHC8 |  |  | 2163 | 0.255 | 0.2739 | No |
| 292 | ARL4A |  |  | 2167 | 0.255 | 0.2741 | No |
| 293 | ARHGEF39 |  |  | 2168 | 0.255 | 0.2750 | No |
| 294 | C2orf69 |  |  | 2183 | 0.254 | 0.2722 | No |
| 295 | ANXA5 |  |  | 2187 | 0.253 | 0.2724 | No |
| 296 | SEPHS1 |  |  | 2188 | 0.253 | 0.2734 | No |
| 297 | PKM |  |  | 2199 | 0.252 | 0.2716 | No |
| 298 | PTPRE |  |  | 2265 | -0.255 | 0.2551 | No |
| 299 | ZNF24 |  |  | 2310 | -0.260 | 0.2442 | No |
| 300 | RAB13 |  |  | 2381 | -0.268 | 0.2264 | No |
| 301 | PSME1 |  |  | 2413 | -0.272 | 0.2191 | No |
| 302 | RNF181 |  |  | 2442 | -0.276 | 0.2126 | No |
| 303 | LRRC58 |  |  | 2447 | -0.276 | 0.2126 | No |
| 304 | IFT52 |  |  | 2472 | -0.280 | 0.2072 | No |
| 305 | MID1 |  |  | 2486 | -0.283 | 0.2048 | No |
| 306 | CDK19 |  |  | 2543 | -0.292 | 0.1908 | No |
| 307 | HMGN3 |  |  | 2561 | -0.295 | 0.1874 | No |
| 308 | TOB1 |  |  | 2580 | -0.298 | 0.1837 | No |
| 309 | RPL3 |  |  | 2613 | -0.303 | 0.1762 | No |
| 310 | SRGAP2B |  |  | 2638 | -0.307 | 0.1709 | No |
| 311 | HSP90B1 |  |  | 2648 | -0.308 | 0.1697 | No |
| 312 | C12orf57 |  |  | 2698 | -0.316 | 0.1577 | No |
| 313 | PCM1 |  |  | 2715 | -0.320 | 0.1546 | No |
| 314 | IVD |  |  | 2723 | -0.321 | 0.1539 | No |
| 315 | CCDC167 |  |  | 2753 | -0.327 | 0.1474 | No |
| 316 | GAS2L3 |  |  | 2755 | -0.327 | 0.1484 | No |
| 317 | NGLY1 |  |  | 2766 | -0.330 | 0.1469 | No |
| 318 | HSPA13 |  |  | 2773 | -0.331 | 0.1466 | No |
| 319 | NES |  |  | 2803 | -0.335 | 0.1401 | No |
| 320 | METTL7A |  |  | 2818 | -0.339 | 0.1376 | No |
| 321 | BSG |  |  | 2844 | -0.343 | 0.1322 | No |
| 322 | ZNF43 |  |  | 2872 | -0.348 | 0.1262 | No |
| 323 | FIGN |  |  | 2879 | -0.351 | 0.1260 | No |
| 324 | KDM4A |  |  | 2900 | -0.355 | 0.1219 | No |
| 325 | RSRC1 |  |  | 2966 | -0.367 | 0.1058 | No |
| 326 | SERF2 |  |  | 2975 | -0.368 | 0.1051 | No |
| 327 | SNX3 |  |  | 2987 | -0.370 | 0.1036 | No |
| 328 | NUDCD2 |  |  | 3003 | -0.373 | 0.1010 | No |
| 329 | TBL1XR1 |  |  | 3032 | -0.380 | 0.0949 | No |
| 330 | ABAT |  |  | 3075 | -0.391 | 0.0850 | No |
| 331 | TAF7 |  |  | 3109 | -0.402 | 0.0777 | No |
| 332 | AGL |  |  | 3134 | -0.410 | 0.0728 | No |
| 333 | TMSB4X |  |  | 3141 | -0.412 | 0.0728 | No |
| 334 | PIK3R3 |  |  | 3151 | -0.414 | 0.0719 | No |
| 335 | DZIP3 |  |  | 3159 | -0.416 | 0.0716 | No |
| 336 | HMGN5 |  |  | 3173 | -0.421 | 0.0698 | No |
| 337 | PON2 |  |  | 3216 | -0.432 | 0.0601 | No |
| 338 | ANP32A |  |  | 3217 | -0.432 | 0.0618 | No |
| 339 | RGS3 |  |  | 3260 | -0.443 | 0.0521 | No |
| 340 | DECR1 |  |  | 3261 | -0.444 | 0.0539 | No |
| 341 | NDUFAF3 |  |  | 3286 | -0.452 | 0.0491 | No |
| 342 | HDAC9 |  |  | 3288 | -0.453 | 0.0506 | No |
| 343 | SMARCA1 |  |  | 3293 | -0.455 | 0.0513 | No |
| 344 | DAZAP2 |  |  | 3319 | -0.463 | 0.0463 | No |
| 345 | HSPA5 |  |  | 3335 | -0.468 | 0.0441 | No |
| 346 | ASRGL1 |  |  | 3362 | -0.483 | 0.0389 | No |
| 347 | MARCKS |  |  | 3369 | -0.487 | 0.0392 | No |
| 348 | GLCCI1 |  |  | 3444 | -0.517 | 0.0212 | No |
| 349 | NDUFA2 |  |  | 3445 | -0.517 | 0.0232 | No |
| 350 | COX17 |  |  | 3461 | -0.524 | 0.0212 | No |
| 351 | CDK6 |  |  | 3466 | -0.526 | 0.0221 | No |
| 352 | QKI |  |  | 3467 | -0.526 | 0.0241 | No |
| 353 | H3F3A |  |  | 3471 | -0.528 | 0.0254 | No |
| 354 | JAM2 |  |  | 3477 | -0.532 | 0.0261 | No |
| 355 | ARL6IP1 |  |  | 3574 | -0.586 | 0.0024 | No |
| 356 | ZEB1 |  |  | 3589 | -0.598 | 0.0010 | No |
| 357 | HNRNPH2 |  |  | 3595 | -0.601 | 0.0019 | No |
| 358 | TFDP2 |  |  | 3637 | -0.634 | -0.0067 | No |
| 359 | SMIM14 |  |  | 3654 | -0.651 | -0.0085 | No |
| 360 | TMEM256 |  |  | 3673 | -0.662 | -0.0108 | No |
| 361 | HIGD2A |  |  | 3679 | -0.668 | -0.0096 | No |
| 362 | MPPED2 |  |  | 3701 | -0.685 | -0.0126 | No |
| 363 | LCORL |  |  | 3718 | -0.698 | -0.0142 | No |
| 364 | PET100 |  |  | 3727 | -0.706 | -0.0137 | No |
| 365 | TGIF1 |  |  | 3728 | -0.707 | -0.0109 | No |
| 366 | FAT1 |  |  | 3730 | -0.708 | -0.0085 | No |
| 367 | COX6C |  |  | 3792 | -0.782 | -0.0219 | No |
| 368 | MSI2 |  |  | 3816 | -0.802 | -0.0250 | No |
| 369 | MYEF2 |  |  | 3818 | -0.807 | -0.0222 | No |
| 370 | NLGN4X |  |  | 3823 | -0.810 | -0.0202 | No |
| 371 | PAIP2 |  |  | 3830 | -0.821 | -0.0186 | No |
| 372 | RHOBTB3 |  |  | 3838 | -0.829 | -0.0173 | No |
| 373 | SALL1 |  |  | 3863 | -0.855 | -0.0205 | No |
| 374 | CHD7 |  |  | 3880 | -0.904 | -0.0213 | No |
| 375 | SORT1 |  |  | 3891 | -0.929 | -0.0205 | No |
| 376 | ZFAND6 |  |  | 3904 | -0.952 | -0.0200 | No |
| 377 | GPX4 |  |  | 3915 | -0.986 | -0.0189 | No |
| 378 | SEMA5A |  |  | 3921 | -1.006 | -0.0164 | No |
| 379 | BCHE |  |  | 3926 | -1.012 | -0.0136 | No |
| 380 | WWTR1 |  |  | 3936 | -1.027 | -0.0121 | No |
| 381 | RAB3IP |  |  | 3940 | -1.037 | -0.0089 | No |
| 382 | GNG11 |  |  | 3952 | -1.062 | -0.0078 | No |
| 383 | SOX2 |  |  | 3953 | -1.069 | -0.0037 | No |
| 384 | HES6 |  |  | 3961 | -1.092 | -0.0014 | No |
| 385 | FRMD4B |  |  | 3972 | -1.116 | 0.0002 | No |
| 386 | ZBTB20 |  |  | 4024 | -1.407 | -0.0081 | No |
| 387 | ASCL1 |  |  | 4061 | -1.762 | -0.0110 | No |
| 388 | CKB |  |  | 4075 | -2.149 | -0.0063 | No |
| 389 | LMO1 |  |  | 4093 | -3.174 | 0.0013 | No |
Table: GSEA details [plain text format]

  

Fig 2: RICHARDS\_NOWAKOWSKI\_SCIENCE\_2017\_MGE\_IPC1\_UPREG: Random ES distribution      
 Gene set null distribution of ES for **RICHARDS\_NOWAKOWSKI\_SCIENCE\_2017\_MGE\_IPC1\_UPREG**
